# Gene Expression as a Molecular Component of the Physiological Response to Mechanical Shell Damage in the Pacific Oyster *Magallana gigas*

**DOI:** 10.3390/vetsci13101051

**Published:** 2026-10-07

**Authors:** Ekaterina S. Kladchenko, Daria S. Lavrichenko, Viktoria A. Uppe, Elizaveta A. Bogacheva, Ekaterina A. Vodiasova, Elina S. Chelebieva

**Affiliations:** A.O. Kovalevsky Institute of Biology of the Southern Seas of RAS, Moscow 119991, Russia; lavrichenko_daria@ibss-ras.ru (D.S.L.); uppeviktoria@ibss-ras.ru (V.A.U.); liza.bogacheva@ibss-ras.ru (E.A.B.); e.vodiasova@ibss-ras.ru (E.A.V.); e.chelebieva@ibss-ras.ru (E.S.C.)

**Keywords:** *Magallana gigas*, shell damage, shell repair, energy metabolism, antioxidant defense, innate immunity

## Abstract

Shell damage is common in bivalves and initiates repair processes that may affect the physiological state of the organism. We examined temporal changes in the expression of genes related to energy metabolism, antioxidant defense, and innate immunity in the mantle and gills of the Pacific oyster *Magallana gigas* during 14 days following experimental shell damage. In the mantle, *PDK1* expression increased on day 1, while on day 7 both *PDK1* and *IDH* expression decreased. The latter period was also characterized by decreased expression of the antioxidant defense-related genes *Mn-SOD* and *GPx* and the immune-related gene *TNF-2*. In the gills, fewer changes were observed: *IDH* and *IL-17-3* expression decreased on day 1, whereas *Toll-1* expression decreased on day 14. No significant differences were detected for the remaining analyzed genes. Thus, significant gene expression differences between shell-damaged and control oysters were detected at different sampling times and showed distinct patterns in the mantle and gills. The mantle showed an early change in the expression of an energy metabolism-related gene, followed by concurrent changes in the expression of genes associated with energy metabolism, antioxidant defense, and immune regulation on day 7. By day 14, no significant differences in the expression of the analyzed mantle genes were detected between shell-damaged and control oysters. The more limited and temporally distinct changes observed in the gills indicate that the gene expression response to shell damage differed between the mantle and gills.

## Simple Summary

Shell damage is common in bivalves and can affect their physiological condition during shell repair. In this study, we examined the response of Pacific oysters to experimental shell damage over 14 days. We measured the expression of genes related to energy metabolism, antioxidant defense, and immune responses in the mantle and gills. The mantle is directly involved in shell formation, whereas the gills are not. In the mantle, changes in gene expression were detected on the first day after shell damage, with more pronounced changes observed on day 7. By day 14, the expression of the analyzed genes in damaged oysters did not differ significantly from that in control oysters. Fewer changes were detected in the gills, and they occurred at different sampling times. The gills showed fewer significant gene expression differences, which were detected at different sampling times than those observed in the mantle.

## 1. Introduction

The shell is a key structural and functional component of the bivalve body, protecting soft tissues from predators, mechanical damage, and adverse environmental conditions [1,2]. It is a complex organomineral biocomposite composed primarily of calcium carbonate and an organic matrix [3,4,5,6]. Shell calcification is influenced by environmental conditions, including temperature, oxygen availability, and seawater carbonate chemistry [7,8]. Once formed, the integrity of these structures may be compromised by predators, mechanical disturbance, and bioeroding organisms [9,10,11]. Boring sponges of the genera *Cliona* and *Pione* and polychaetes of the genus *Polydora* are among the bioeroders that have been demonstrated to cause substantial damage to cultured bivalves [10,12,13]. The production of networks of tunnels and cavities within the shell by these organisms compromises its protective function and progressively erodes the shell structure. In response to such damage, bivalves initiate processes of shell repair and biomineralization [10].

Calcification and shell repair are notably energetically demanding processes. In the pearl oyster *Pinctada fucata martensii*, studies have demonstrated that shell repair is associated with alterations in glycolysis, the tricarboxylic acid cycle, pyruvate metabolism, and amino sugar metabolism [14]. Alterations in carbohydrate metabolism have also been reported in *Mizuhopecten yessoensis* following shell damage caused by the boring polychaete *Polydora* [15]. Consequently, the energy that would otherwise be available for other energy-demanding physiological functions is reduced [10,16,17]. In accordance with these energetic trade-offs, bivalves affected by shell-damaging epibionts may exhibit reduced growth, lower soft-tissue mass, and decreased nutritional value [11,18,19]. The energetic demands of shell repair have also been demonstrated to affect physiological defense mechanisms, particularly immune and antioxidant responses. As indicated in the relevant literature, alterations in immune and antioxidant defenses have been documented in bivalves exposed to other environmental stressors [20,21,22]. The maintenance of these defense systems assumes particular importance in circumstances where shell integrity is compromised, and protection against environmental stressors is reduced [23].

Bivalves depend on innate immune defenses that involve pattern-recognition receptors, cytokines, and hemocyte-mediated cellular responses [24,25,26,27]. In *P. fucata martensii*, shell damage was associated with pronounced changes in the expression of immune-related genes [14], while several immune-related genes, including *TNF*, showed altered expression following shell damage in *Mytilus* [28]. It is noteworthy that such damage may also have implications for redox homeostasis. In *M. gigas* affected by the boring sponge *Pione vastifica*, increased production of reactive oxygen species and oxidative damage have been reported, together with tissue-specific changes in the activity and expression of components of the antioxidant defense system [29,30,31,32]. Collectively, these findings indicate that shell damage is associated with changes in genes related to energy metabolism, immune regulation, and antioxidant defense. However, previous studies have largely examined these processes separately or in the context of prolonged exposure to bioeroding organisms. The temporal gene expression response to acute mechanical shell damage therefore remains poorly characterized.

The present study examined changes in the expression of genes associated with energy metabolism (*PEPCK*, *PK*, *PDK1*, *IDH*, and *MDH*), antioxidant defense (*Mn-SOD*, *Cu/Zn-SOD*, *CAT*, and *GPx*), and innate immunity (*TNF-1*, *TNF-2*, *Toll-1*, *IL-17-3*, and *IL-17-6*) in *M. gigas* during 14 days following experimental mechanical shell damage. Gene expression was assessed in the mantle, which is directly involved in shell formation and repair, and in the gills, which are not directly involved in shell deposition. Comparison of these tissues allowed us to distinguish gene expression changes occurring in the tissue directly associated with shell formation from those occurring in a tissue not directly involved in this process.

## 2. Materials and Methods

### 2.1. Animal Collection and Maintenance

Triploid Pacific oysters (5 years old; 90.7 ± 7.1 g; shell length 17.2 ± 2.1 cm) were obtained in March from Mariculture Ltd. (Sevastopol Bay, Black Sea, Sevastopol, Ukraine), an aquaculture facility near Sevastopol (44.616014, 33.502248). Prior to the experiment, the oysters were visually inspected, and only individuals with intact shells and no visible signs of previous shell damage were selected. Sex was not determined. The oysters were transported to the laboratory in insulated containers devoid of water, a strategy employed to preserve hydration and mitigate the deleterious effects of transportation-related stress. The tanks were filled with filtered artificial seawater, and the oysters were maintained at a salinity of 18 ‰, pH 7.8, temperature of 15 ± 0.5 °C, under continuous aeration and a 12 h light:12 h dark photoperiod. Prior to the experiment, the oysters were acclimated to laboratory conditions for a period of two weeks (oxygen content: 8 mg·L^−1^, temperature of 15 ± 1 °C, and salinity of 18 ± 1). The seawater in the tank was replaced on a daily basis. During the acclimation process, oysters were provided with a daily diet consisting of a mixture of microalgae species, including *Tetraselmis viridis* and *Chlorella vulgaris* f. *suboblonga*, at a biomass ratio of 1:1. To ensure the maintenance of a density of approximately 4 × 10^5^ cells·mL^−1^, an algae blend was added to the experimental tanks. The feeding regimens of the shell-damaged and control groups were matched. Following acclimation, oysters assigned to each experimental condition were randomly distributed between 2 replicate tanks, with 15 individuals per tank. Thus, two independent tanks were maintained for each experimental condition. At each sampling time, 5 oysters were randomly sampled from each replicate tank, ensuring equal representation of both tanks in the biological sample (*n* = 10 per condition). This balanced design was used to minimize potential tank-specific variation.

### 2.2. Experimental Shell Damage

The experimental shell damage was produced by creating multiple small circular defects in both shell valves. Thirty defects, approximately 1–2 mm in diameter, were created on each valve using a small electric dental handpiece equipped with a round dental bur with a working-head diameter of 1.6 mm. The defects were distributed within a shell region extending from the hinge toward the adjacent shell area and occupying approximately one-third of the surface area of each valve. This estimate refers to the shell region over which the defects were distributed rather than to the cumulative surface area of the individual defects. Drilling was limited to the superficial shell layers and stopped before the inner shell surface was reached. The integrity of the inner shell surface was confirmed by visual inspection after drilling. The entire shell-damage procedure lasted no more than 5 min. During the procedure, control oysters were kept adjacent to the shell-damaged oysters and were subjected to the same handling, duration of air exposure, ambient drilling noise, and environmental vibration, but their shells were not drilled. After the procedure, oysters from both groups were returned to aerated tanks.

### 2.3. Tissue Collection

At each sampling time, 5 oysters were sampled from each replicate tank, giving 10 oysters per experimental condition (*n* = 10) on days 1, 7, and 14. These time points were selected based on previous studies in *M. gigas*, which reported early changes in calcium uptake within 24 h and the onset of shell mineral deposition during the following 1–2 weeks [33,34]. At each sampling time, 10 oysters from the shell-damaged group and 10 oysters from the control group were sampled (*n* = 10 per group). Consequently, a total of 60 oysters were utilized for the collection of tissue samples. Mantle and gill tissues were collected from each individual specimen. In oysters with shell damage, mantle tissue was excised from the region adjacent to the injured shell area. In oysters without damage, the corresponding mantle region was sampled for comparison. Tissue samples were immediately frozen in liquid nitrogen and stored at −80 °C until RNA extraction.

### 2.4. Gene Expression Analysis by RT-qPCR

Total RNA was extracted from *M. gigas* mantle and gill tissues using the RNeasy Mini Kit (Qiagen, Hilden, Germany), following the manufacturer’s protocol. The extracted RNA was treated with DNase I at 37 °C for 15 min. Following this treatment, the RNA was resuspended in 50 µL of nuclease-free water. The integrity of RNA was confirmed through agarose gel electrophoresis with ethidium bromide staining, and the concentration was measured using a Qubit 4.0 fluorometer (Therma Fisher Scientific, Waltham, MA, USA). The average RNA concentration was 132.02 ng/µL; samples with an RNA concentration of less than 85 ng/µL were re-extracted. Subsequently, cDNA was synthesized using the MMLV kit (Evrogen, Moscow, Russia), following the manufacturer’s protocol. The reverse transcription process was conducted in a final volume of 15 μL, comprising 500 ng of purified RNA. The expression of genes associated with energy metabolism (*PEPCK*, *PK*, *PDK1*, *MDH*, and *IDH*), antioxidant defense (*CAT*, *Mn-SOD*, *Cu/Zn-SOD*, and *GPx*), and innate immunity (*TNF-1*, *TNF-2*, *Toll-1*, *IL-17-3*, and *IL-17-6*) was analyzed. Initially, β-actin and EF-1α were evaluated as potential reference genes. The stability of their expression across experimental groups and sampling points was assessed using BestKeeper v.1 [35]. EF-1α showed greater expression stability (the mean Ct was 18.57 with standard deviation 0.74) and was therefore selected as the reference gene for normalization, whereas β-actin showed higher variability across treatments (SD > 1.00) leading to its exclusion in analysis. Primer sequences and corresponding references are provided in Table 1. Quantitative real-time PCR analysis was performed on a CFX 96 Real-Time PCR System (Bio-Rad Laboratories, Hercules, CA, USA) and BioMaster SYBR Blue qPCR mix (Biolabmix, Novosibirsk, Russia). The reaction mixture (total volume 25 µL) contained 0.5 µL of cDNA, 0.5 µM of each primer, qPCR master mix, and nuclease-free water. Thermal cycling consisted of an initial denaturation at 95 °C for 30 s, followed by 40 cycles of 95 °C for 5 s, 55 °C for 20 s, and 72 °C for 10 s, with a final extension at 72 °C for 7 min. A melting curve analysis (55–95 °C, ramp rate 0.5 °C/s) was included to verify amplicon specificity. All reactions were performed in technical triplicate, and no-template controls were included in each run. Quantification cycle (Cq) values were determined using CFX 96 software (v. 3.1). Amplification efficiencies were evaluated using standard curves generated from serial dilutions of cDNA. The resulting amplification efficiencies are presented in Table 1. Relative gene expression was calculated using the 2^−ΔΔCT^ [36] and normalized to *EF-1α.*

### 2.5. Statistical Analyses

Statistical analyses were performed in R (version 4.6.3) [45]. Relative-expression values, calculated using the 2^−ΔΔCT^ method, were log2-transformed prior to statistical analysis. Normality and homogeneity of variances were assessed using the Shapiro–Wilk and Levene tests, respectively. For each gene, individual relative-expression values calculated using the 2^−ΔΔCt^ method were analyzed using a three-way mixed ANOVA with experimental condition and sampling time as between-subject factors and tissue as a within-subject factor. Main effects and interactions were evaluated, followed by planned comparisons between shell-damaged and control oysters within each tissue and sampling time. *p*-values from all planned comparisons across genes, tissues, and sampling times were adjusted jointly using the Benjamini–Hochberg procedure, with adjusted *p* < 0.05 considered statistically significant.

## 3. Results

The three-way mixed ANOVA identified significant effects of experimental condition on the expression of *IDH*, *IL-17-3*, *PDK1*, *TNF-2*, and *Toll-1* (Table 2). A significant condition × tissue interaction was detected for IDH, whereas no significant condition × time or condition × time × tissue interactions were observed for the analyzed genes.

Significant differences between shell-damaged and control oysters in the expression of energy metabolism-related genes were detected at different sampling times and showed distinct patterns in the mantle and gills (Figure 1). In the mantle, *PDK1* expression was significantly increased on day 1 compared with the control group, whereas on day 7 its expression was significantly decreased. *IDH* expression in the mantle was also significantly decreased on day 7. No significant differences in mantle *PEPCK*, *PK*, or *MDH* expression were detected at any sampling point. By day 14, the expression levels of all analyzed energy metabolism genes in the mantle did not differ significantly from the control. In the gills, *IDH* expression was significantly decreased on day 1, whereas no significant differences were detected on days 7 and 14. The expression of *PEPCK*, *PK*, *PDK1*, and *MDH* in the gills did not differ significantly between the shell-damaged and control groups at any sampling point.

Changes in the expression of antioxidant defense-related genes were detected only in the mantle on day 7 after shell damage (Figure 2). At this sampling point, *Mn-SOD* and *GPx* expression were significantly decreased compared with the control group, whereas *Cu/Zn-SOD* and *CAT* expression did not differ significantly. No significant differences in the expression of the analyzed antioxidant defense-related genes were detected in the mantle on days 1 and 14. In the gills, the expression of *Mn-SOD*, *Cu/Zn-SOD*, *CAT*, and *GPx* did not differ significantly between the shell-damaged and control groups at any sampling point.

Significant differences in the expression of immune-related genes were detected at different sampling times and showed distinct patterns in the mantle and gills (Figure 3). In the mantle, *TNF-2* expression was significantly decreased on day 7, whereas no significant differences were detected on days 1 and 14. The expression of *TNF-1*, *Toll-1*, *IL-17-3*, and *IL-17-6* in the mantle did not differ significantly between the shell-damaged and control groups at any sampling point. In the gills, *IL-17-3* expression was significantly decreased on day 1, while *Toll-1* expression was significantly decreased on day 14. No significant differences in *IL-17-3* expression were detected on days 7 and 14, and *Toll-1* expression did not differ from the control on days 1 and 7. No significant differences in *TNF-1*, *TNF-2*, and *IL-17-6* expression in the gills were detected throughout the experiment.

## 4. Discussion

The present study investigated the effects of experimental shell damage on the expression of genes related to energy metabolism, antioxidant defense, and innate immunity in *M. gigas*. The most significant alterations were detected in the mantle, where genes belonging to all three functional categories exhibited altered expression (Figure 4). In contrast, a smaller number of examined genes were affected in the gills. Gene expression changes represent an important molecular component of the physiological response to stress and can provide insight into the regulation of physiological processes.

### 4.1. Energy Metabolism

Changes in energy metabolism-related gene expression were most evident in the mantle during the first week after shell damage. *PDK1* was upregulated on day 1 but downregulated on day 7, when *IDH* was also downregulated. In the gills, only *IDH* was significantly downregulated on day 1. No significant differences in PEPCK, PK, or MDH expression were detected between shell-damaged and control oysters at any sampling time. Overall, the response was largely confined to *PDK1* and *IDH*.

The initial upregulation of *PDK1* in the mantle indicates a potential alteration in the regulation of pyruvate metabolism subsequent to shell damage. *PDK1* has been shown to phosphorylate and inhibit the pyruvate dehydrogenase complex [44], thereby regulating the conversion of pyruvate to acetyl-CoA and its subsequent entry into oxidative metabolism. However, it is important to note that *PDK1* transcript levels alone cannot be used to infer pyruvate dehydrogenase activity or the direction of metabolic flux. Changes in energy metabolism during the process of shell repair have also been reported in *Pinctada fucata martensii*. In this species, shell damage altered the expression of genes associated with glycolysis, the tricarboxylic acid cycle, and pyruvate metabolism [14].

*IDH* catalyzes the conversion of isocitrate to 2-oxoglutarate in the tricarboxylic acid cycle [46]. Its downregulation may therefore reflect altered regulation of this component of energy metabolism, although transcript levels alone cannot be used to infer enzyme activity or metabolic flux. The early decrease in *IDH* expression in the gills further suggests that the response to shell damage was not confined to the mantle. However, the absence of significant changes in *PEPCK*, *PK*, *PDK1*, and *MDH* suggests a relatively limited response of the examined energy metabolism-related genes in the gills.

By day 7, no differences in the examined energy metabolism-related genes were detected in the gills. In the mantle, however, the initial upregulation of *PDK1* was followed by its downregulation together with *IDH*. This temporal pattern suggests that the expression of these genes in the mantle changes during the post-damage period. Such a pattern is consistent with the concept of energy homeostasis, whereby increased energetic demands for maintenance and repair may redistribute a limited energy budget among competing physiological functions [16,17]. By day 14, no significant differences in the expression of the examined genes were detected between shell-damaged and control oysters. This finding does not establish that expression had returned to control levels.

Previous studies in *M. gigas* provide additional context for this temporal pattern. Changes in calcium uptake have been reported within the first day after shell damage, whereas shell mineral deposition was observed during the following 1–2 weeks [33,34]. In addition, changes in the expression of stress-related genes in *M. gigas* were pronounced during the first week of prolonged stress and decreased by day 14 [47]. Thus, the sampling points examined here may correspond to different phases of the post-damage response, with day 7 representing the period when the largest number of significant gene expression changes was detected in the mantle and day 14 a later phase in which no significant differences were detected for the examined mantle genes (Figure 4). However, because shell deposition and calcification were not directly assessed, these phases remain hypothetical and should not be considered experimentally defined stages of shell repair.

### 4.2. Antioxidant Response

Changes in antioxidant-related gene expression were less pronounced than those observed for energy metabolism and were restricted to the mantle on day 7 after shell damage. *Mn-SOD* and *GPx* were downregulated, whereas no significant differences were detected in *Cu/Zn-SOD* and *CAT* expression throughout the experiment. Superoxide dismutases catalyze the dismutation of superoxide anions to H_2_O_2_, while *CAT* and *GPx* are involved in peroxide detoxification. *Mn-SOD* primarily protects mitochondria against superoxide, whereas *Cu/Zn-SOD* functions predominantly outside the mitochondrial matrix [48,49,50]. The downregulation of *Mn-SOD* and *GPx*, with no significant differences detected in *Cu/Zn-SOD* and *CAT*, suggests selective regulation of individual components of the antioxidant system rather than a general suppression of antioxidant defense.

Differential responses of antioxidant components have also been reported in other bivalve studies. In *M. gigas* exposed to cadmium, *CAT* and *GPx* expression peaked on day 3 and subsequently declined, whereas *SOD* expression reached its maximum on day 7 [50]. Different temporal patterns of *SOD*, *CAT*, and *GPx* activities were also observed following tissue injury and implantation procedures associated with pearl production in *Pteria sterna* [51]. In *M. galloprovincialis*, antioxidant enzyme responses differed between the gills and mantle and varied with exposure duration [52]. In *M. gigas* affected by the boring sponge *Pione vastifica*, increased ROS production and DNA damage in hemocytes were not accompanied by changes in *SOD*, *CAT*, or *GPx* activity in the mantle [29]. In another study, increased lipid peroxidation was accompanied by increased *CAT* activity and decreased *GPx* activity in the mantle [31]. The antioxidant response also varied with the severity of infestation and the mantle region examined [30,32]. Together, these studies show that individual components of the antioxidant system respond differently depending on the nature and duration of the stressor and, in cases of shell damage, on the severity and location of the damage.

The downregulation of *Mn-SOD* and *GPx* on day 7 coincided with reduced *PDK1* and *IDH* expression in the mantle, indicating that changes in antioxidant defense- and energy metabolism-related genes occurred at the same sampling time. This temporal correspondence is consistent with the concept of a limited energy budget, whereby increased energetic demands for maintenance and repair may alter resource allocation among competing physiological functions [16,17]. The concurrent downregulation of *Mn-SOD* and *GPx* with *PDK1* and *IDH* may therefore reflect concurrent changes in the expression of genes associated with antioxidant defense and energy metabolism following shell damage.

### 4.3. Immune Response

Changes in immune-related gene expression following shell damage were limited, with significant differences detected at different sampling times in the mantle and gills. In the gills, *IL-17-3* was downregulated on day 1 and *Toll-1* on day 14, whereas *TNF-2* was downregulated in the mantle on day 7. No significant differences in *TNF-1* and *IL-17-6* were detected throughout the experiment. *IL-17* and *TNF* are cytokines involved in the regulation of immune and inflammatory responses, whereas Toll-like receptors participate in immune recognition and downstream innate immune signaling [53,54,55,56]. Members of the *IL-17* and *TNF* families in *M. gigas* may differ in their regulation and functional roles [53,54,56]. The changes in *IL-17-3* and *TNF-2*, together with the absence of significant differences in *IL-17-6* and *TNF-1*, suggest selective regulation of individual components of innate immunity rather than a general suppression of the immune response. Selective changes in immune-related gene expression have also been reported during shell repair in other bivalves. In *Pinctada fucata martensii*, shell damage induced changes in genes associated with immune responses during the repair process [14]. Similarly, shell damage in *Mytilus coruscus* was accompanied by changes in the expression of several immune-related genes, including *TNF* [28].

Significant differences in immune-related gene expression were detected at different sampling times in the mantle and gills. The early downregulation of *IL-17-3* in the gills coincided with that of *IDH*, indicating concurrent changes in immune- and energy metabolism-related markers in biomineralization. In the mantle, *TNF-2* was downregulated on day 7 together with *PDK1*, *IDH*, *Mn-SOD*, and *GPx*, coinciding with the most pronounced changes in the energy metabolism- and antioxidant-related genes examined in this study. *TNF-2* is involved in the regulation of antibacterial and hemocyte responses [56,57,58]. The coincidence of these responses does not necessarily imply a direct regulatory relationship among them but indicates that changes in genes associated with several physiological processes occurred during the same period after shell damage. Such changes may occur in the context of resource allocation among competing physiological functions, as proposed within the framework of energy homeostasis [16,17]. In contrast, *Toll-1* was downregulated in the gills on day 14, when no changes were detected in the energy metabolism- or antioxidant-related genes examined, and may therefore represent a later change in immune regulation.

## 5. Conclusions

Experimental shell damage in *M. gigas* was associated with selective changes in the expression of genes related to energy metabolism, antioxidant defense, and immune regulation, which were detected at different sampling times in the mantle and gills. The most pronounced response was observed in the mantle on day 7, where changes in *PDK1* and *IDH* expression coincided with decreased expression of *Mn-SOD*, *GPx*, and *TNF-2*. In contrast, the gills showed a different temporal pattern, with significant changes in *IDH* and *IL-17-3* on day 1 and *Toll-1* on day 14. Most of the other genes examined did not differ significantly between shell-damaged and control oysters. Overall, these results indicate that shell damage induces selective changes in the expression of the examined genes rather than a generalized response, with distinct temporal patterns in the mantle and gills. The concurrent gene expression changes observed in the mantle on day 7 indicate that genes associated with energy metabolism, antioxidant defense, and immune regulation responded during the same period after shell damage.

## Figures and Tables

**Figure 1:**
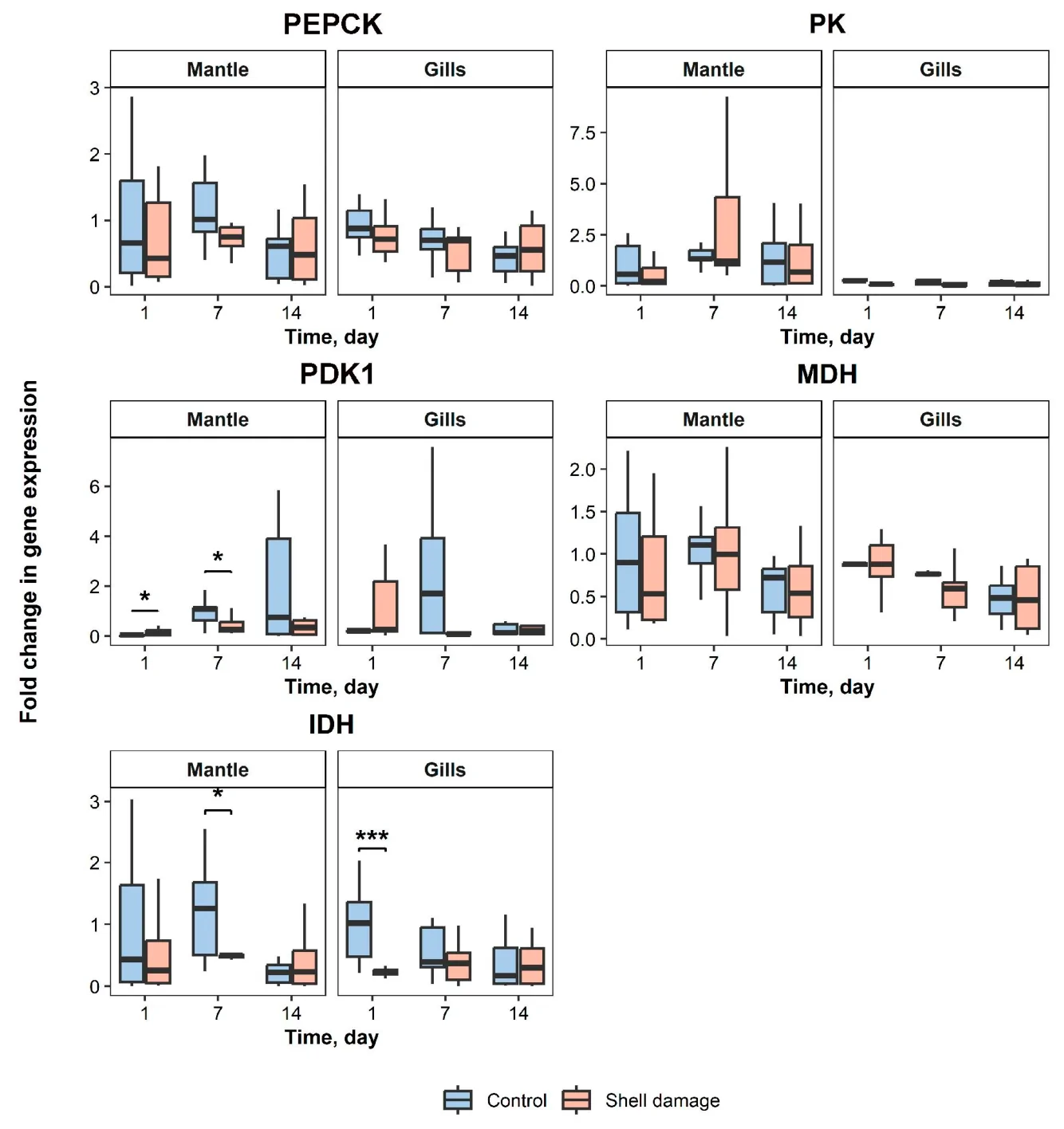
Expression of energy metabolism-related genes in the mantle and gills of control and shell-damaged Pacific oysters. Relative expression levels of *PEPCK*, *PDK1*, *IDH*, *MDH*, and *PK* were determined in the mantle and gills of control and shell-damaged oysters on days 1, 7, and 14 after experimental shell damage. Data are presented as boxplots showing the median and interquartile range; whiskers extend to 1.5 × the interquartile range. Asterisks indicate significant differences between shell-damaged and control oysters within the same tissue and sampling day after adjustment for multiple comparisons (*n* = 10 per group; * *p* < 0.05, *** *p* < 0.001).

**Figure 2:**
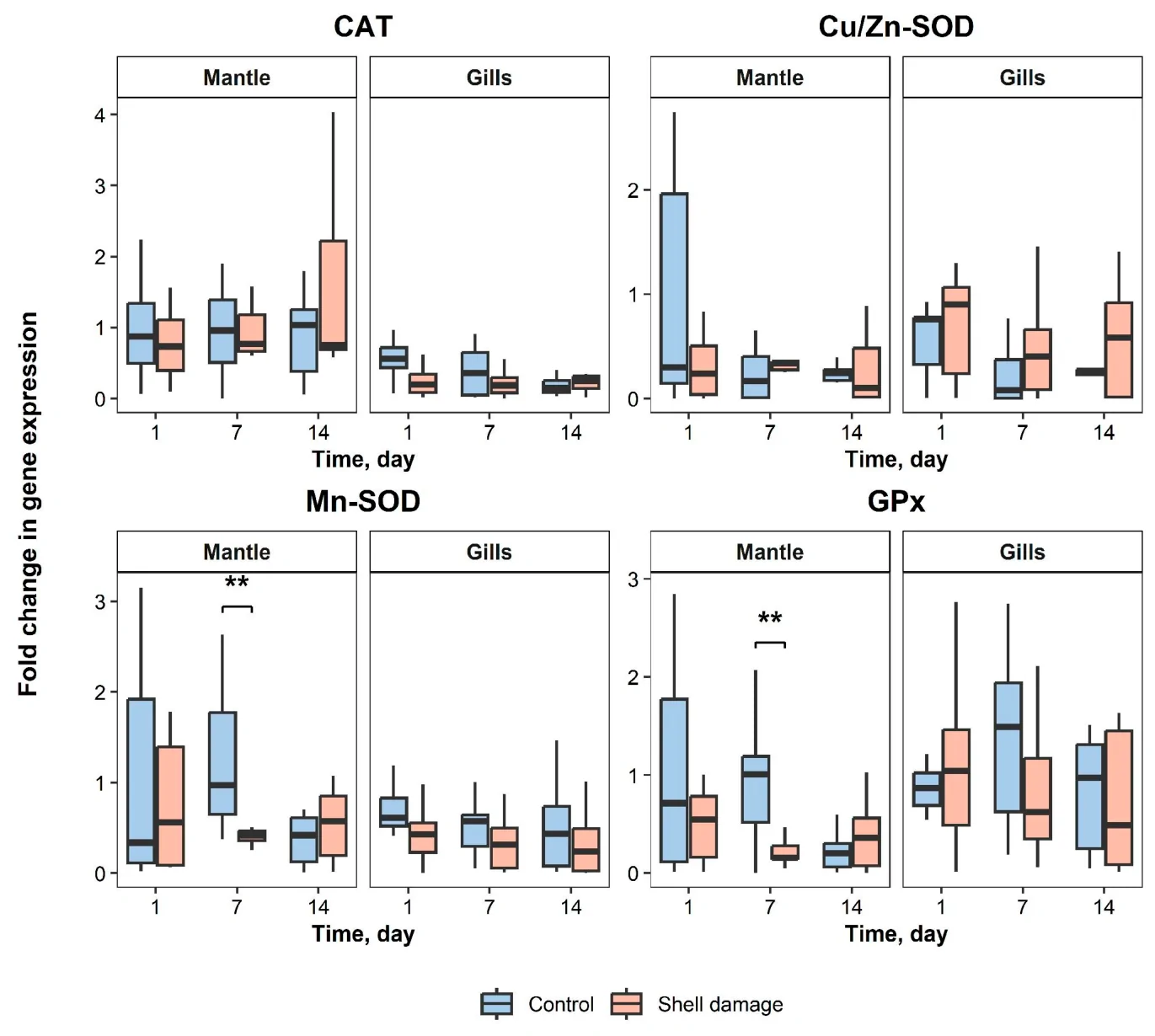
Expression of antioxidant-related genes in the mantle and gills of control and shell-damaged Pacific oysters. Relative expression levels of *CAT*, *Cu/Zn-SOD*, *Mn-SOD*, and *GPx* were determined in the mantle and gills of control and shell-damaged oysters on days 1, 7, and 14 after experimental shell damage. Data are presented as boxplots showing the median and interquartile range; whiskers extend to 1.5 × the interquartile range. Asterisks indicate significant differences between shell-damaged and control oysters within the same tissue and sampling day after adjustment for multiple comparisons (*n* = 10 per group; ** *p* < 0.01).

**Figure 3:**
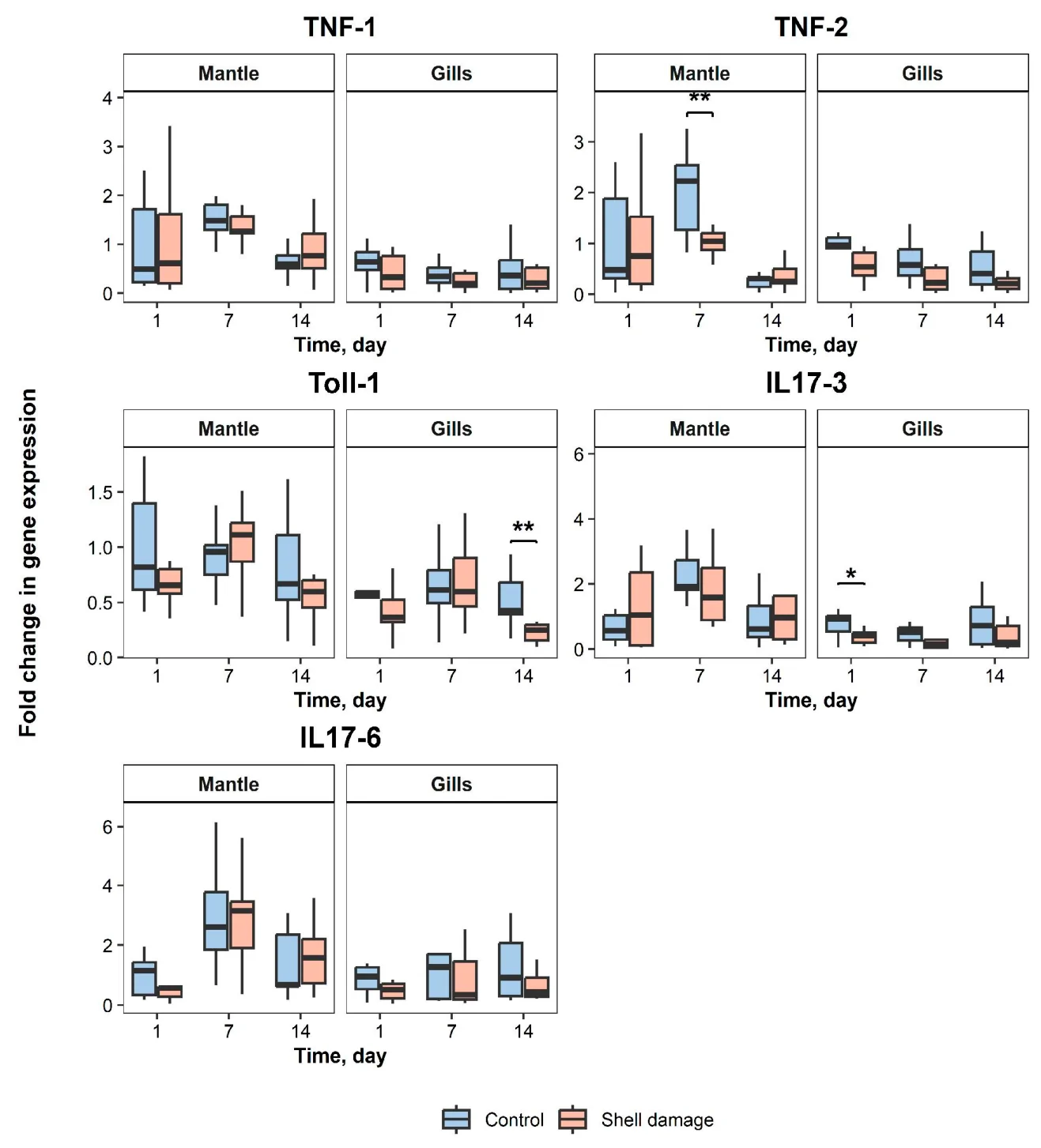
Expression of immune-related genes in the mantle and gills of control and shell-damaged Pacific oysters. Relative expression levels of *TNF-1*, *TNF-2*, *Toll-1*, *IL-17-3*, and *IL-17-6* were determined in the mantle and gills of control and shell-damaged oysters on days 1, 7, and 14 after experimental shell damage. Data are presented as boxplots showing the median and interquartile range; whiskers extend to 1.5 × the interquartile range. Asterisks indicate significant differences between shell-damaged and control oysters within the same tissue and sampling day after adjustment for multiple comparisons (*n* = 10 per group; * *p* < 0.05, ** *p* < 0.01).

**Figure 4:**
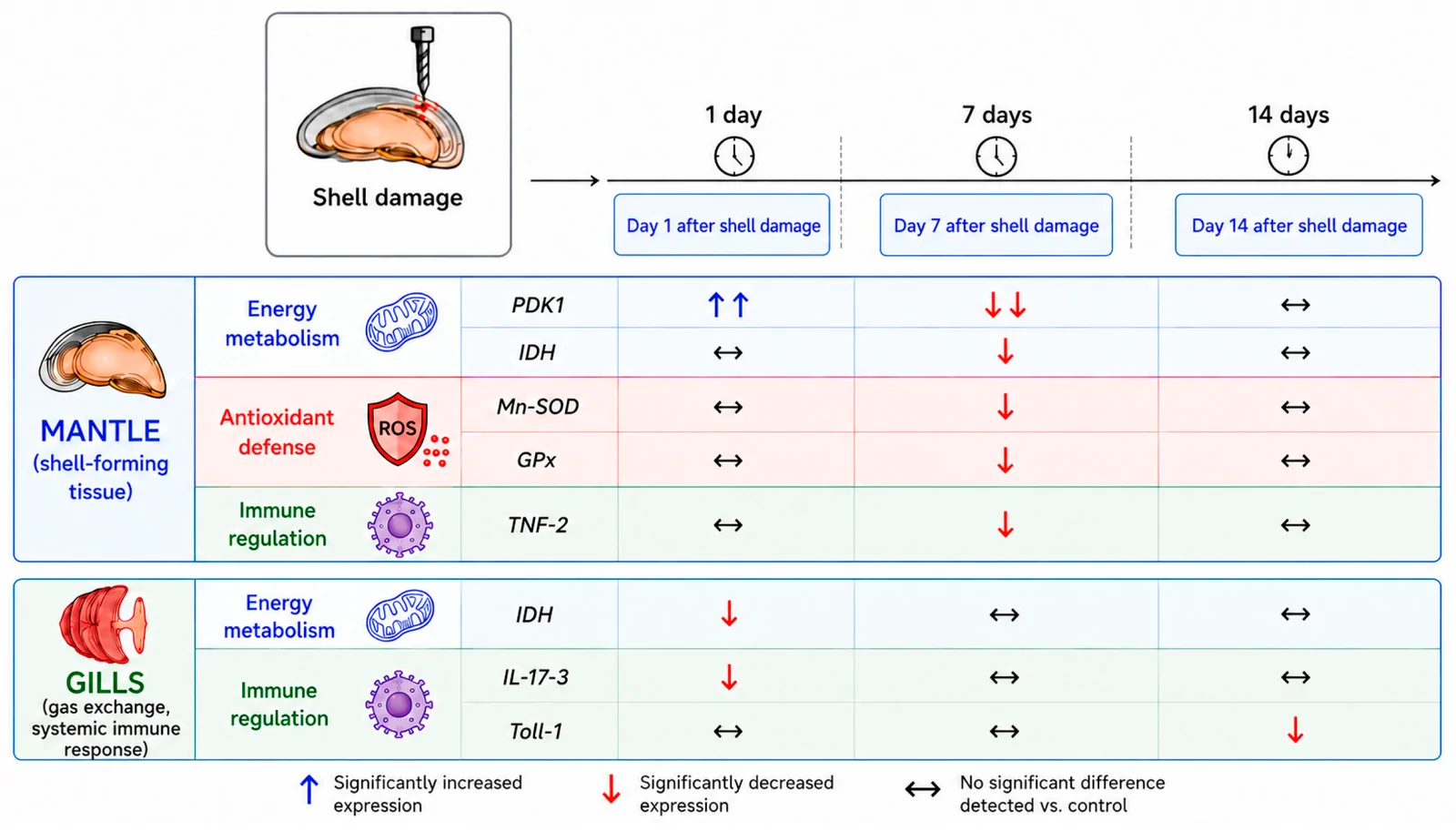
Temporal patterns of gene expression responses to experimental shell damage in the mantle and gills of *Magallana gigas*. The schematic summarizes significant changes in the expression of genes related to energy metabolism, antioxidant defense, and innate immunity at 1, 7, and 14 days after shell damage. Upward and downward arrows indicate significantly increased and decreased expression, respectively, in shell-damaged oysters relative to controls; horizontal arrows indicate no significant difference between groups.

**Table 1:** Primer sequences of target and reference genes for RT-qPCR.

Gene Name	Sequence	Efficiency (%)	Function	Source
*IL-17-3*	F: TCACTACCAACTGAACTACGACCG R: TGCGACAGAGCCTGAGAACC	103.0	Interleukin-17 (proinflammatory cytokine)	[37]
*IL-17-6*	F: CCATTTGTCCGACCTACCGT R: CCACCCTGCGTAGAACCATC	98.5	Interleukin-17 (proinflammatory cytokine)	[37]
*TNF-1*	F: CTTCTCGTCTGCGGCTTCTTT R: CAGGGCTGCGGTCTTTCC	102.0	Tumor necrosis factor (proinflammatory cytokine)	[37]
*TNF-2*	F: CCACTACGCCCACTAAGAAACTG R: AAGCGACCATTGCGAAACTCCAT	100.5	Tumor necrosis factor (proinflammatory cytokine)	[37]
*Toll-1*	F: AAGTTCTCGTGAAGTCCCATCC R: ATACAGGCGTCTCGTAACCATC	100.0	Toll-like receptor	[38]
*CAT*	F: TTCGTCATATCGGGTTTACTTCTG R: CCTTGTCACGTCCTGCCATT	99.0	Catalase (oxidative stress defense)	[39]
*Cu/Zn-SOD*	F: CCAGAGGATCACGAGAGGC R: GCGTTTCCGGTCGTCTT	107.0	Cu/Zn superoxide dismutase (oxidative stress defense)	[40]
*Mn-SOD*	F: ACAAAGTCAATCAGTGCCCT R: CCATTGCCTCTGCCAGT	97.5	Mn superoxide dismutase (oxidative stress defense)	[41]
*GPx*	F: ATCGAACGCTGCACCA R: AGCTCCGTCGCATTGT	93.1	Glutathione peroxidase (oxidative stress defense)	[41]
*IDH*	F: CTCACGGGGACCAGTACAAAG R: AGCAGGTATGGGCAAAAGCA	109.0	Isocitrate dehydrogenase	[42]
*MDH*	F: TCTATGGCGGTTCCGTCTG R: GTCGTCGATGGGCAAGTC	102.0	Malate dehydrogenase	[43]
*PDK1*	F: CAATGGAAGGCTACGGGACA R: TCCAAGTGGTGCTGCTTGAC	108.5	Pyruvate dehydrogenase kinase 1	[44]
*PEPCK*	F: GGGTGGATGAGCATGGGAAA R: CTGAACAGCTCGTCCCACTT	109	Phosphoenolpyruvate carboxykinase	[44]
*PK*	F: GTACCGGCCTGATTGTGTGA R: TGTCGCCGCTGTTGATGTAT	93.4	Pyruvate kinase	[44]
*EF-1α*	F: AGTCACCAAGGCTGCACAGAAAG R: TCCGACGTATTTCTTTGCGATGT	99.8	Elongation factor 1-alpha (reference gene)	[44]
*β-actin*	F: AGTACCCCATTGAACACGG R: TGGCGGGAGCGTTGAA	100.8	β-actin (reference gene)	[41]

**Table 2:** Three-way mixed ANOVA results for treatment-related effects on gene expression.

Gene	Condition	Condition × Time	Condition × Tissue	Condition × Time × Tissue
*CAT*	F(1, 42) = 0.32; *p* = 0.575	F(2, 42) = 0.54; *p* = 0.588	F(1, 42) = 2.85; *p* = 0.099	F(2, 42) = 0.02; *p* = 0.983
*Cu/Zn-SOD*	F(1, 35) = 1.59; *p* = 0.215	F(2, 35) = 0.63; *p* = 0.536	F(1, 35) = 0.15; *p* = 0.703	F(2, 35) = 0.34; *p* = 0.716
*GPx*	**F(1, 43) = 4.03; *p* = 0.051**	F(2, 43) = 0.71; *p* = 0.498	F(1, 43) = 0.28; *p* = 0.599	F(2, 43) = 0.05; *p* = 0.951
*IDH*	**F(1, 47) = 4.20; *p* = 0.046**	F(2, 47) = 0.27; *p* = 0.764	**F(1, 47) = 4.24; *p* = 0.045**	F(2, 47) = 0.04; *p* = 0.964
*IL-17-3*	**F(1, 44) = 4.97; *p* = 0.031**	F(2, 44) = 0.77; *p* = 0.469	F(1, 44) = 0.83; *p* = 0.366	F(2, 44) = 0.02; *p* = 0.976
*IL-17-6*	F(1, 42) = 0.38; *p* = 0.541	F(2, 42) = 0.33; *p* = 0.720	F(1, 42) = 0.60; *p* = 0.443	F(2, 42) = 0.10; *p* = 0.909
*MDH*	F(1, 48) = 2.93; *p* = 0.094	F(2, 48) = 0.05; *p* = 0.950	F(1, 48) = 0.03; *p* = 0.871	F(2, 48) = 0.06; *p* = 0.940
*Mn-SOD*	F(1, 44) = 2.10; *p* = 0.154	F(2, 44) = 0.86; *p* = 0.428	F(1, 44) = 0.46; *p* = 0.501	F(2, 44) = 0.07; *p* = 0.932
*PDK1*	**F(1, 43) = 4.22; *p* = 0.046**	F(2, 43) = 0.79; *p* = 0.459	F(1, 43) = 0.05; *p* = 0.828	F(2, 43) = 0.96; *p* = 0.389
*PEPCK*	F(1, 48) = 1.18; *p* = 0.282	F(2, 48) = 0.10; *p* = 0.909	F(1, 48) = 0.04; *p* = 0.848	F(2, 48) = 0.03; *p* = 0.972
*PK*	F(1, 47) = 0.18; *p* = 0.674	F(2, 47) = 0.15; *p* = 0.863	F(1, 47) = 2.78; *p* = 0.102	F(2, 47) = 0.89; *p* = 0.419
*TNF-1*	F(1, 45) = 0.90; *p* = 0.349	F(2, 45) = 0.68; *p* = 0.512	F(1, 45) = 1.40; *p* = 0.243	F(2, 45) = 0.46; *p* = 0.632
*TNF-2*	**F(1, 45) = 5.92; *p* = 0.019**	F(2, 45) = 0.43; *p* = 0.653	F(1, 45) = 0.65; *p* = 0.424	F(2, 45) = 0.08; *p* = 0.924
*Toll-1*	**F(1, 45) = 5.71; *p* = 0.021**	F(2, 45) = 2.23; *p* = 0.119	F(1, 45) = 2.60; *p* = 0.114	F(2, 45) = 0.97; *p* = 0.389

Bold values indicate statistically significant differences.

## Data Availability

The data presented in this study are available on request from the corresponding author.

## Abbreviations

The following abbreviations are used in this manuscript:

PDK1	Pyruvate dehydrogenase kinase
IDH	Isocitrate dehydrogenase
SOD	Superoxide dismutase
GPx	Glutathione peroxidase
TNF	Tumor necrosis factor
PEPCK	Phosphoenolpyruvate carboxykinase
PK	Pyruvate kinase
MDH	Malate dehydrogenase
CAT	Catalase
IL	Interleukin
RNA	Ribonucleic acid
cDNA	Complementary DNA
